# Crop production in Russia 2030: Alternative data of the development scenarios

**DOI:** 10.1016/j.dib.2019.105077

**Published:** 2020-01-22

**Authors:** Evgeny Vladimirovich Rudoy, Marina Sergeevna Petukhova, Andrey Fedorovich Petrov, Svetlana Yurievna Kapustyanchik, Inga Nikolaevna Ryumkina, Sergey Vladimirovich Ryumkin

**Affiliations:** Branch Center for Forecasting and Monitoring of Scientific and Technological Development of Agroindustrial Complex, Novosibirsk State Agrarian University, Novosibirsk, Russian Federation

**Keywords:** Crop production, Seed growing, Organic farming, Scenarios, Gross yield, Productivity, Sown area, Forecasting

## Abstract

This article reveals the main indicators of scientific and technological development (gross yield, yield, acreage and average annual prices for crop products) of the crop industry in the long term. Using these indicators, two development scenarios “Technological adaptation” and “Technological breakthrough” of the crop production sector until 2030 were identified. Scenarios for the development of the crop production industry in Russia until 2030 were constructed by means of a correlation and regression analysis. The objectivity of using the obtained regression equations is confirmed by the strong relationship between factors and gross yield. These factors are the basis for determining the gross yield of each crop in 2030. The article also presents the forecast of export volumes of agricultural crops in Russia. Predicted indicators of the Russian crop industry indicate an increase in the volume of seed and organic products.

**Table:** Specifications Table

Subject	*Economy, Agriculture, Crop production*
Specific subject area	*Agronomy, Organic farming, Seed growing*
Type of data	*Text, table, figures and graphs*
How data were acquired	*Information base of researches is created with use of official* *Statistical data of the Federal state statistics service: the content of the official website of Rosstat* (http://www.gks.ru/), *statistical collections, data reviews and reports of the Ministry of Agriculture of the Russian Federation* [[4], [5], [6]].
Data format	*Raw and analyzed*
Parameters for data collection	*The parameters for data collection are the main indicators (gross harvest, yield, acreage and average annual crop prices) in the long term*.
Description of data collection	*Models of scenarios of development of crop production industry “Technological adaptation” and “Technological breakthrough” with indicators of development of grain production and production of oil crops, sugar beet, potatoes and vegetables of the open ground till 2030. The data for the research were calculated using the formulas of the linear regression equation and the correlation coefficient*. *In calculations software packages are used: Microsoft Office Excel, StatSoft STATISTICA 12*
Data source location	*Novosibirsk State Agrarian University*, *Novosibirsk, Russian Federation*
Data accessibility	*The raw data files are provided in the Data in Brief Dataverse*, https://doi.org/10.7910/DVN/MOSFP5. *All other data is with this article*
Related research article	*This article is a continuation of the research “Crop production in Russia 2030: Scenarios based on data from the scientific and technological development of the sector” by E.V. Rudoi, S.V. Ryumkin, M.S. Petukhova (Data in brief 25 (2019)*). https://doi.org/10.1016/j.dib.2019.103980

**Table:** 

**Value of the Data** • The regression equations make it possible to calculate the forecast gross yield of crops in the country for two different scenarios; • These data can also be used to develop strategic documents in the crop sector; • The data allow us to build a probable picture of the future scientific and technological development of the Russian crop production industry.

## 1 Data

**Fig. 1:**
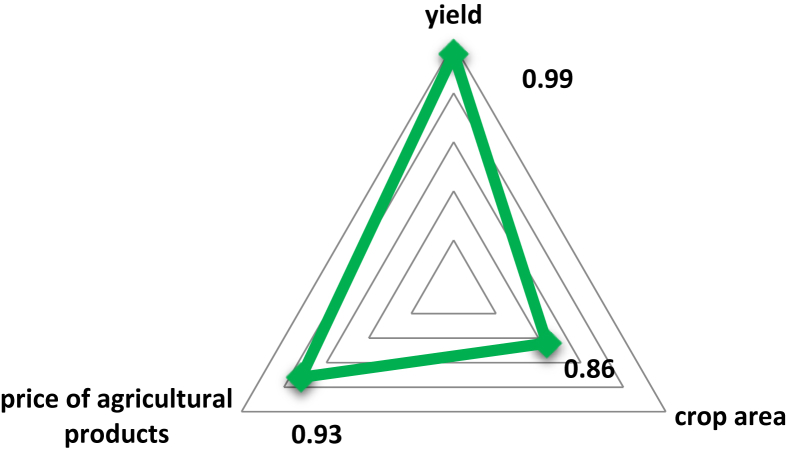
The diagram of the distribution of correlation coefficients between factors of production and the indicator of gross harvest.

**Fig. 2:**
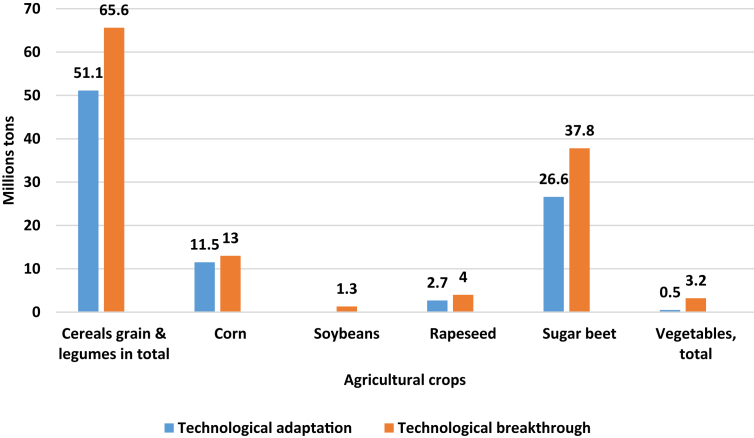
Export of major crop crops under the proposed scenarios (in millions of tons).

**Fig. 3:**
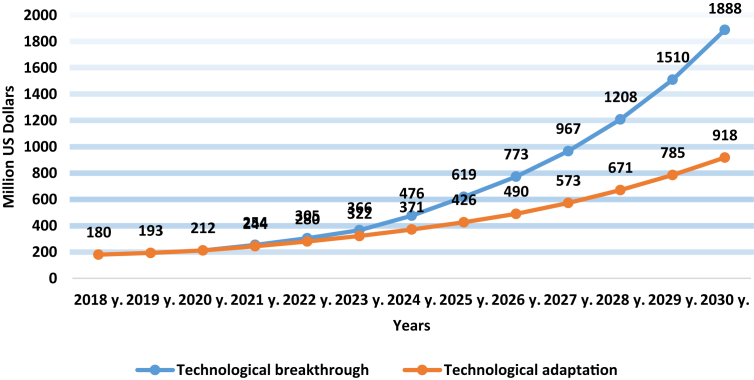
The projected growth of the organic products market in the Russian Federation under the scenarios “Technological adaptation” and “Technological breakthrough”.

The data set in this article allows you to see simulated scenarios for the development of the crop industry with their indicators until 2030. Table 1. Results of the regression analysis. Fig. 1. The diagram of the distribution of correlation coefficients between factors of production and the indicator of gross harvest. Table 2. Forecast change in crop production volume and structure in 2030. Fig. 2. Export of major crop crops under the proposed scenarios (in millions of tons). Table 3. Forecast need for seeds of agricultural crops for each of the scenarios in 2030 in the Russian Federation. Table 4. Forecast of organic agriculture development indicators according to the scenarios “Technological adaptation” and “Technological breakthrough” in the Russian Federation by 2030. Fig. 3. The projected growth of the organic products market in the Russian Federation under the scenarios “Technological adaptation” and “Technological breakthrough”.

**Table 1:** Results of the regression analysis.

Crop	The resulting regression equation
Grain	у = 5,54х_1_ + 0,002х_2_ – 0,001х_3_ – 96,8
Wheat	у = 3,42х_1_ + 0,001х_2_ – 0,0006х_3_ – 46,5
Corn	у = 0,15х_1_ + 0,003х_2_ + 0,0003х_3_ – 5,7
Rye	у = 0,22х_1_ + 0,002х_2_ – 0,00005х_3_ – 3,8
Buckwheat	у = 0,13х_1_ + 0,0007х_2_ + 0,000002х_3_ – 1,1
Rice	у = 0,01х_1_ + 0,003х_2_ + 0,000008х_3_ + 0,3
Barley	у = 0,9х_1_ + 0,002х_2_ + 0,00006х_3_ – 22,4
Soybeans	у = 0,1х_1_ + 0,001х_2_ – 0,000001х_3_ – 1,4
Sunflower	у = 0,8х_1_ + 0,0006х_2_ + 0,000005х_3_ – 6,7
Rapeseed	у = 0,03х_1_ + 0,003х_2_ + 0,00009х_3_ – 3,5
Sugar beet	у = 0,1х_1_ + 0,003х_2_ + 0,0002х_3_ – 29,8
Potatoes	у = 0,23х_1_ + 0,01х_2_ – 0,0001х_3_ – 26,2
Open grounds vegetables	у = 0,06х_1_ + 0,01х_2_ – 0,000005х_3_ – 7,9

**Table 2:** Forecast change in crop production volume and structure in 2030.

Crop	2017 г.		Scenario “Technological adaptation”		Scenario “Technological breakthrough”	
	Million tons	%	Million tons	%	Million tons	%
**Cereals grain & legumes in total, including**	**134,1**	**100,0**	**146,7**	**100**	**161,2**	**100**
Wheat	85,5	63,8	88,6	60,4	97,2	60,3
Corn	12,1	9,0	24,5	16,7	26,0	16,1
Barley	20,6	15,4	15,8	10,8	25,1	15,6
Rye	2,5	1,9	1,8	1,2	2,9	1,8
Buckwheat	1,5	1,1	1,5	1,0	1,8	1,1
Rice	1	0,7	1,4	1,0	2,1	1,3
other	10,9	8,1	13,1	8,9	6,1	3,8
**Oil crops, total**	**15,4**	**100,0**	**23,8**	**100**	**26,7**	**100**
Sunflower	9,6	62,3	14,1	59,2	15,2	56,9
Soybeans	3,6	23,4	4,1	17,2	5,8	21,7
Rapeseed	1,5	9,7	4,7	19,8	6,0	22,5
other	0,7	4,5	0,9	3,8	1,5	5,6
**Sugar beet**	**48,2**	–	**66,6**	–	**77,8**	–
**Potatoes**	**29,6**	–	**30,0**	–	**40,3**	–
**Vegetables, total**	**16,35**	**100,0**	**17,6**	**100**	**21**	**100**
open ground	15,4	94,2	16,1	91,5	17,7	84,3
protected ground	0,95	5,8	1,5	8,5	3,3	15,7

Bold text indicates the total values for groups of agricultural crops cultivated in Russia. Within each group, the specific weight of each culture in the final value of the group is represented. Exceptions are potatoes and sugar beets.

**Table 3:** Forecast need for seeds of agricultural crops for each of the scenarios in 2030 in the Russian Federation.

Crops	Actually sown in 2017	Scenario «Technological adaptation»		Scenario “Technological breakthrough”	
	thousand tons	thousand tons	Growth rate, in %	thousand tons	Growth rate, in %
**Cereals grain & legumes, in total (without corn):**	**9149,1**	**9515,0**	104,0	**10,064,0**	110,0
Wheat	5738,7	5968,2	104,0	6771,7	118,0
Barley	1680,1	1764,1	105,0	1932,1	115,0
Oats	682,4	699,5	102,5	682,4	100,0
Buckwheat	110,4	113,7	103,0	119,2	108,0
Rice	48,4	50,8	105,0	53,2	110,0
other	889,1	918,7	103,3	505,4	56,8
**Corn**	**87,2**	**91,6**	**105,0**	**95,9**	**110,0**
**Fiber flax**	**3,8**	**4,0**	**105,3**	**4,2**	**110,5**
**Sunflower**	**36,3**	**38,1**	**105,0**	**39,9**	**109,9**
**Soybeans**	**294,2**	**308,9**	**105,0**	**323,6**	**110,0**
**Rapeseed**	**7,7**	**8,1**	**105,2**	**8,5**	**110,4**
**Sugar beet**	**4,1**	**4,3**	**104,9**	**4,5**	**109,8**
**Potatoes**	**743,1**	**780,0**	**105,0**	**817,4**	**110,0**
**Total**	**10,325,5**	**10,750,0**	**104,1**	**11,358,0**	**110,0**

**Table 4:** Forecast of organic agriculture development indicators according to the scenarios “Technological adaptation” and “Technological breakthrough” in the Russian Federation by 2030.^1^

Indicators	2016 year	Scenario «Technological adaptation»	Scenario “Technological breakthrough”
The number of certified organic producers, units	80	450	1500
The volume of organic products market, in million US dollars	160	918	1888
Area of certified land, million hectares	0,25	1,44	4,5
Consumption of organic products per capita, in US dollars	1,1	45	70

1 The forecast is based on expert assessments.

## 2 Experimental design, materials, and methods

In previous researches, proposed two scenarios for the scientific and technological development of the Russian crop industry until 2030 [1]. The major factor in predicting the scientific and technological development of the industry is the yield, which depends on the level of development of agricultural technologies. In the calculations, it was assumed that the scale of technical and technological modernization of production is estimated by means of predicted yield growth. In the base regression models, which became the basis for the “Technological adaptation” scenario, yield forecasting was carried out by continuing its existing trend, and for the “Technological breakthrough” scenario, the possibility of its growth was taken into account by using modern high-performance machines, equipment and technologies in production [2,3].

An important direction for the development of the crop growing industry for Russia is organic farming. Below are the forecast data on the main indicators of the development of organic farming in Russia, taking into account global trends, as well as data on the market volumes of organic products for each of the scenarios.

The calculation of forecast indicators was based on the constructed regression equations, where the result was the gross yield of crops, and the factors - yield, sown area and average selling prices (Table 1).

The objectivity of using the obtained regression equations is confirmed by the strong relationship between factors and gross yield.

The greatest impact on the result has crop yields. A similar dependence (with a slight deviation) is true for each of the cultures presented.

### 2.1 Crop production

One of the main indicators of the crop industry is the production volume (Table 2).

In the “Technological adaptation” scenario, by 2030 it is planned to increase the gross harvest of grain and legumes by 9% compared to 2017 to 146.7 million tons, due to a slight increase in yield and stagnation of the sown area, an increase of which is impossible due to the inability of most agricultural producers to process large areas (lack of technical equipment). The structure of production will not change significantly: the largest share will belong to wheat - 60.4% [4,5].

The growth in grain production will be due to both an increase in domestic consumption from 80.8 to 95.6 million tons (including 60 million tons for feed purposes), and due to an increase in external demand for Russian grain from 42.5 million tons in 2017 up to 50 million tons in 2030.

The high profitability of maize production and the increase in demand for maize from foreign countries will lead to a significant increase in its share in grain production from 9% in 2017 to 16.7 and 16.1% by 2030, respectively, in each of the scenarios.

Buckwheat harvest in 2017 amounted to 1.5 million tons, which is twice the demand of the domestic market, which is currently over-saturated [6]. In this regard, in two scenarios it is planned to reduce gross culture collection up to 2025 (in order to stabilize the market situation), and further it is expected to grow to 1.5 and 1.8 million tons respectively by 2030.

Slight changes will occur in the structure of oilseeds: the proportion of soybeans will decrease by 6.2%, and rapeseed will increase by 10.1% due to the growth of biofuel production in foreign countries. At the same time, the gross yield of oilseeds will increase by 55% - up to 23.8 million tons.

The sugar beet market will grow at a slow pace, in 2030 the gross harvest will be 66.6 million tons. For domestic consumption of sugar, it is required to process 40 million tons, the remaining volume will be exported to other countries.

The production of vegetables by 2030 will amount to 17.6 million tons («Technological adaptation») and 21 million tons («Technological breakthrough»), which will cover most of the country's population's needs for products, which was 16.35 million tons, in 2017. More than 8.5% of vegetables will be grown in protected ground, the growth of which will be about 5% annually due to modernization of old and introduction of new greenhouse complexes.

The “Technological breakthrough” scenario predicts an increase in gross grain harvest to 161.2 million tons. Domestic grain consumption will not change, as in the “Technological adaptation” scenario, and the remaining 65.6 million tons will be exported as food and raw materials for biofuel production, the demand for which will gradually increase in Europe and the USA.

Gross harvest of oilseeds will amount to 26.7 million tons. The proportion of rapeseed will increase by 12.8% to 6 million tons in 2030 due to an increase in sown area and crop yields. This scenario will make it possible to understand that soybeans and rapeseed are strategically important crops for Russia, the demand for which is constantly growing abroad. These crops will help expand the country's export structure.

Sugar beet production in 2030 will amount to 77.8 million tons, which is 61% higher than in 2017 due to the sustainable development of sown areas and yield growth.

The main reason for the development of fruit and vegetable production until 2030 is the growth of incomes of the population with the shift of consumer demand in favor of fresh vegetables and greens. The annual growth rate of new greenhouse areas will average 15% (over the past 5 years, the growth rate has been 13–14%). The share of protected ground vegetables will increase from 5.8 to 15.7%. Some products (3.2 million tons) will be exported (Egypt, China, India, etc.). Since the beginning of 2017, there has been a sharp increase in the export (3.7 times or 29%) of Russian tomatoes and cucumbers abroad, which will continue to grow in the medium and long term, but in less significant volumes.

### 2.2 Export crop production

Prediction of crop export volumes was carried out by subtracting the volume of domestic consumption from the gross yield.

Assessing and analyzing data on the volume of exports, in addition to increasing the share of Russian grain and leguminous crops in the current largest markets, it is planned to actively promote countries where Russian products are represented in small volumes or not at all: African markets and South Asian countries, including India and Pakistan.

Due to the fact that in the medium term it is planned to increase grain consumption for biofuel by 12%, and the main demand is forecasted for it abroad, then, therefore, the export of Russian grain crops will increase. At the same time, the demand for grain crops as food will decrease, and for fodder purposes it will increase, due to the projected increase in livestock production. Among these crops, there is corn. For example, in the United States, in the medium term, it is planned that corn production will prevail over wheat (the economic effect of growing corn is higher than that of other crops, given that the price of corn has been growing in recent years). Farmers are increasingly favouring more cost-effective corn.

The increase in soybean export growth is due to the widespread use of biofuels in the world and the growing population of China, which is the main exporter of soybeans.

### 2.3 Selection and seed growing

Over the past five years, there has been a steady increase in sown areas under winter and spring wheat. Thanks to export deliveries, the mixed feed production and the development of deep processing of grain, the demand for products and services of the grain complex will increase until 2030. The growth in the “Technological adaptation” scenario will be 4%, in the “Technological breakthrough” scenario - up to 10%.

According to the “Technological adaptation” scenario, an increase in the production of major crops (wheat, barley, rice) and oilseeds (corn, flax, sunflower, soybeans, rape) is expected, and, therefore, the average growth rate in seed needs for these crops will be 4–5% annually.

The “Technological breakthrough” scenario also predicts an increase in seed needs by an average of 10%. The highest growth is expected in seeds of barley, rice, corn, fiber-flax, soybean, rapeseed, potatoes.

### 2.4 Organic farming

It is rather difficult to predict the indicators of the development of organic farming, due to the fact that this direction in Russia is in an embryonic state. The volume of this domestic market is very small. Agricultural producers with European organic agricultural product certificates mainly export their products as raw materials to European countries. The cost of organic grain is on average higher in the world by 40–50%.

Low demand for organic products and distrust of agricultural producers to expand this market is the main factors constraining the development of organic farming in Russia.

The forecast of the National organic Union of Russia assumes an increase in the number of certified companies as a percentage of the world indicator from 0.005% to 1–2%. These data allow to conclude that by 2030 under the scenario “Technological adaptation” this indicator will make 450 units, and under the scenario “Technological breakthrough” will be1500 units.

According to Organic Monitor, the organic products market in Russia will increase to 250 million US dollars by 2020 [7]. It is expected that in the period from 2020 to 2025 the market volume will increase and reach 30% per year, in connection with the adoption of the Federal Law “On Production and Circulation of Organic Products.”

Since 2022, the “Technological breakthrough” scenario is projected to be gradually separated from the “Technological adaptation” scenario due to an increase in domestic demand for organic products caused by the global trend of a healthy lifestyle, which is gaining more and more popularity and influence. In this regard, by 2030, the expenses of Russian residents on organic products will increase and amount to 45 US dollars per capita in the first scenario and 70 US dollars per capita in the second scenario. For reference comparison in 2016 in Canada this indicator was 91 $, in the USA - 101 $. As a result, by 2030, the growth of the organic product market will be according to scenario forecasts “Technological adaptation” will be 918 million US dollars and “Technological breakthrough” will be 1888 million US dollars.
